# Association Between Physician Depressive Symptoms and Medical Errors: A Systematic Review and Meta-analysis

**DOI:** 10.1001/jamanetworkopen.2019.16097

**Published:** 2019-11-27

**Authors:** Karina Pereira-Lima, Douglas A. Mata, Sonia R. Loureiro, José A. Crippa, Lívia M. Bolsoni, Srijan Sen

**Affiliations:** 1 Department of Psychiatry, University of Michigan Medical School, Ann Arbor; 2 Department of Psychiatry, Federal University of São Paulo, São Paulo, Brazil; 3 Department of Pathology, Memorial Sloan Kettering Cancer Center, New York, New York; 4 Department of Neuroscience and Behavior, University of São Paulo, Ribeirão Preto, São Paulo, Brazil

## Abstract

**Importance:**

Depression is highly prevalent among physicians and has been associated with increased risk of medical errors. However, questions regarding the magnitude and temporal direction of these associations remain open in recent literature.

**Objective:**

To provide summary relative risk (RR) estimates for the associations between physician depressive symptoms and medical errors.

**Data Sources:**

A systematic search of Embase, ERIC, PubMed, PsycINFO, Scopus, and Web of Science was performed from database inception to December 31, 2018.

**Study Selection:**

Peer-reviewed empirical studies that reported on a valid measure of physician depressive symptoms associated with perceived or observed medical errors were included. No language restrictions were applied.

**Data Extraction and Synthesis:**

Study characteristics and RR estimates were extracted from each article. Estimates were pooled using random-effects meta-analysis. Differences by study-level characteristics were estimated using subgroup meta-analysis and metaregression. The Preferred Reporting Items for Systematic Reviews and Meta-analyses (PRISMA) guideline was followed.

**Main Outcomes and Measures:**

Relative risk estimates for the associations between physician depressive symptoms and medical errors.

**Results:**

In total, 11 studies involving 21 517 physicians were included. Data were extracted from 7 longitudinal studies (64%; with 5595 individuals) and 4 cross-sectional studies (36%; with 15 922 individuals). The overall RR for medical errors among physicians with a positive screening for depression was 1.95 (95% CI, 1.63-2.33), with high heterogeneity across the studies (χ^2^ = 49.91; *P* < .001; *I*^2^ = 82%; τ^2^ = 0.06). Among the variables assessed, study design explained the most heterogeneity across studies, with lower RR estimates associated with medical errors in longitudinal studies (RR, 1.62; 95% CI, 1.43-1.84; χ^2^ = 5.77; *P* = .33; *I*^2^ = 13%; τ^2^ < 0.01) and higher RR estimates in cross-sectional studies (RR, 2.51; 95% CI, 2.20-2.83; χ^2^ = 5.44; *P* = .14; *I*^2^ = 45%; τ^2^ < 0.01). Similar to the results for the meta-analysis of physician depressive symptoms associated with subsequent medical errors, the meta-analysis of 4 longitudinal studies (involving 4462 individuals) found that medical errors associated with subsequent depressive symptoms had a pooled RR of 1.67 (95% CI, 1.48-1.87; χ^2^ = 1.85; *P* = .60; *I*^2^ = 0%; τ^2^ = 0), suggesting that the association between physician depressive symptoms and medical errors is bidirectional.

**Conclusions and Relevance:**

Results of this study suggest that physicians with a positive screening for depressive symptoms are at higher risk for medical errors. Further research is needed to evaluate whether interventions to reduce physician depressive symptoms could play a role in mitigating medical errors and thus improving physician well-being and patient care.

## Key Points

**Question:**

What are the magnitude and direction of associations between physician depressive symptoms and medical errors?

**Findings:**

In this systematic review and meta-analysis of 11 studies involving 21 517 physicians, physicians with a positive screening for depression were highly likely to report medical errors. Examination of longitudinal studies demonstrated that the association between physician depressive symptoms and medical errors is bidirectional.

**Meaning:**

This study found that physician depressive symptoms were associated with medical errors, highlighting the relevance of physician well-being to health care quality and underscoring the need for systematic efforts to prevent or reduce depressive symptoms among physicians.

## Teaser

This systematic review and meta-analysis examines whether physician depressive symptoms are associated with the risk for perceived or observed medical errors.

## Introduction

Medical errors are a major source of patient harm. Studies estimate that, in the United States, as many as 98 000 to 251 000 hospitalized patients die each year as result of a preventable adverse event.^1,2,3,4^ In addition, medical errors are a major source of morbidity^5^ and account for billions of dollars in financial losses to health care systems every year.^6,7,8,9^

Depressive symptoms are highly prevalent among physicians,^10,11^ and several studies have investigated the associations between physician depressive symptoms and medical errors.^12,13,14,15,16^ Although most studies on physician depressive symptoms and medical errors have identified a substantial association, their results are not unanimous, and questions regarding the direction of these associations remain open in recent literature.^17^

Depressive symptoms have well-established clinical criteria, and a large body of work has demonstrated that depression is a preventable and treatable condition.^18,19,20^ Several studies with physicians have identified potential individual and work environment sources of interventions to prevent the development of depressive symptoms among these professionals,^21,22,23,24^ and although scarce, research on the efficacy of interventions to reduce depressive symptoms in physicians has shown positive results.^25^

Given that depression is preventable and treatable, a reliable estimate of the degree to which physicians with a positive screening for depression are at higher risk for medical errors would be useful. Such an estimate would inform public health decision-making on strategies to improve patient safety and physician well-being. In this systematic review and meta-analysis, we investigated whether physician depressive symptoms were associated with medical errors. We also examined longitudinal studies to investigate the temporal associations between depressive symptoms and medical errors.

## Methods

### Search Strategy and Study Eligibility

Two of us (K.P.-L. and L.M.B.) independently identified cross-sectional and longitudinal studies published before December 31, 2018, that reported on the associations between physician depressive symptoms and perceived or objectively assessed medical errors. We systematically searched Embase, ERIC, PubMed, PsycINFO, Scopus, and Web of Science. In addition, guided by the Preferred Reporting Items for Systematic Reviews and Meta-analyses (PRISMA),^26^ we screened the reference lists of the articles and corresponded with study investigators. The search strategy we used was initially designed by the corresponding author (K.P.-L.), and critical revisions and edits to this design were provided by a multiprofessional team of researchers with expertise in conducting systematic reviews and meta-analyses on physician depression (D.A.M., S.S.) and mental health (S.R.L., J.A.C., S.S.) research. The Ribeirão Preto Medical School Institutional Review Board deemed this study exempt from approval and informed consent because it collected and synthesized nonidentifiable data from previously published studies.

For the database searches, terms related to physicians and depressive symptoms were combined with terms related to medical errors, without language restriction; full details of the search strategy are provided in the eMethods in the Supplement. References identified from database searches were exported to EndNote (Clarivate Analytics). After removal of duplicates, full-text articles were obtained if their abstracts were considered to be eligible by at least 1 of us. Each full-text article was assessed independently for final inclusion in this systematic review and meta-analysis, and disagreements were resolved by consensus (we reached 97% overall agreement [113 of 116 articles; κ = 0.87]). Peer-reviewed studies that reported data on perceived or observed medical errors associated with a valid measure of depressive symptoms in practicing and resident physicians (ie, excluding medical students and other health care professionals) were included. Studies that involved both physicians and other health care professionals were included only if they provided separate data for physicians. To be included, studies did not have to consider the association between physician depressive symptoms and medical errors as their primary outcome of interest.

### Data Extraction and Quality Assessment

Two of us (K.P.-L., L.M.B.) independently extracted the following data from each article using a standardized study form: (1) study information, including geographic location, survey years, research design, sample size, percentage of respondents among eligible participants, and number of institutions included; (2) characteristics of participants, including mean age, percentage of women, specialties, and career level; and (3) outcomes, including depressive symptoms measure, medical errors question interval, method of medical errors assessment, and data for calculating effect size (eg, relative risk [RR], CIs, *P* values). The approach recommended by Zhang and Yu^27^ for converting adjusted odds ratio for RR was used for studies that reported only the results of logistic regression for the associations between physician depressive symptoms and medical errors. Corresponding authors were contacted at least twice when studies did not report enough data to compute the effect size. When studies involved the same population of physicians, only the most comprehensive articles (ie, including those with a greater number of participants or a longer follow-up period) were included.

The methodological quality of the studies was assessed using adapted criteria from the Cochrane Library guidelines.^28^ Studies were considered methodologically strong or weak on the basis of (1) study design (eg, longitudinal indicated strong; cross-sectional, weak), (2) sample size (≥200 participants indicated strong; <200 participants, weak), (3) ascertainment of depressive symptoms measure (sensitivity and specificity >75% indicated strong; sensitivity and specificity ≤75%, weak), (4) representativeness of the sample (≥2 institutions indicated strong; <2 institutions, weak), and (5) descriptive characteristics of participants (reported data on sex, age, specialties, and career level indicated strong; missing information on sex, age, specialties, or career level, weak). Cutoff scores for sample size, representativeness, and descriptive characteristics were based on thresholds used in previous meta-analyses on physician depression,^10,11^ whereas cutoff scores for ascertainment of depressive symptoms were based on well-established psychometric quality criteria for depression questionnaires.^29^ Disagreements regarding quality assessment scores for each individual study were resolved by consensus (with an overall agreement of 98%; κ = 0.96).

### Statistical Analysis

Relative risk estimates of physician depressive symptoms associated with medical errors were calculated by pooling study-specific estimates using random-effects models with generic invariance method to incorporate the heterogeneity of the differences across the studies.

Between-study heterogeneity was measured using standard χ^2^ tests and *I*^2^ statistics (values <25% indicate low; 25%-75%, moderate; and >75%, considerable heterogeneity).^30,31^ Sensitivity analyses were performed by serially excluding each study to determine the implications of individual studies for the pooled RR estimates.

Results from studies grouped according to prespecified study-level characteristics were compared using stratified meta-analysis (for physician career level, specialties included, medical errors question interval, geographic region, depressive symptoms measure, and quality assessment indicators [ie, study design, sample size, ascertainment of the depressive symptoms measure, representativeness of the sample, and descriptive data]) or random-effects metaregression (for year of baseline survey and percentage of women).^32,33^ To gain insight into the direction of the association between depressive symptoms and medical errors, we calculated pooled RR estimates for longitudinal studies that reported (1) results of physician depressive symptoms associated with subsequent medical errors and (2) RR estimates of medical errors associated with subsequent physician depressive symptoms.

Bias secondary to small study effects was investigated using funnel plots and the Egger test.^34,35^ We used R, version 3.2.3 (R Project for Statistical Computing),^36^ with meta^37^ and metafor^38^ packages for all analyses. Statistical tests were 2-sided and used a significance threshold of *P* < .05.

## Results

### Study Characteristics

**Figure 1.**
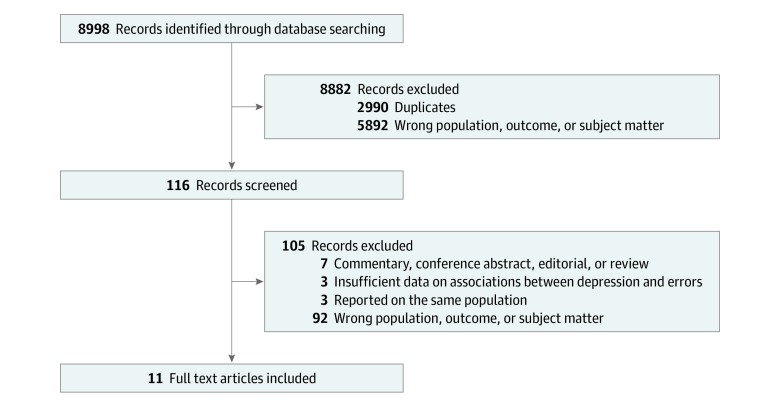
PRISMA Flow Diagram

Eleven studies involving a total of 21 517 physicians were included in this systematic review and meta-analysis (Figure 1). The characteristics of the included studies are summarized in the Table. A total of 7 studies (64%) were longitudinal (involving 5595 individuals)^12,13,14,15,39,40,44^ and 4 (36%) were cross-sectional (involving 15 922 individuals).^16,41,42,43^ Nine studies (82%) took place in the United States,^12,13,14,15,16,40,42,43,44^ 1 (9%) in Japan,^39^ and 1 (9%) in South Korea.^41^ Eight studies (73%) included only training physicians (interns and/or residents),^12,13,14,15,16,40,41,44^ and 3 (27%) recruited physicians from any career level.^39,42,43^ Seven studies (64%) recruited physicians from multiple specialties,^14,15,39,40,41,42,43^ whereas 4 (36%) recruited physicians from a single specialty.^12,13,16,44^ Among these 4 studies, 1 focused on pediatric residents,^12^ 1 on anesthesiology residents,^16^ and 2 on internal medicine residents.^13,44^ The median (interquartile range [IQR]) number of participants per study was 836 (2139). Five studies (46%) assessed depressive symptoms with the 2-item Primary Care Evaluation of Mental Disorders (PRIME-MD-2) questionnaire^13,41,42,43,44^; 3 (27%) used the 9-item Patient Health Questionnaire (PHQ-9)^14,15,40^; 2 (18%) used the Harvard National Depression Screening Day Scale (HANDS)^12,16^; and 1 (9%) used the 5-item World Health Organization Well-being Index (WHO-5).^39^ Sensitivity and specificity commonly reported for these depression instruments are available in eTable 1 in the Supplement.

**Table.** Selected Characteristics of the 11 Included Studies

Source	Country	Baseline Year	Specialties	Participants, No. (%)	Physician Career Level	Age, y (%)	Women, No. (%)	Study Design	Depression Measure (Cutoff Score)	Depression Assessment	Errors	
											Measure	Assessment
Fahrenkopf et al,^12^ 2008	United States	2003	Pediatrics, medicine-pediatrics	101 (50.0)^a^	Training physicians	<30 (61.8)	69.9	Longitudinal	HANDS (≥9)	Baseline	Active surveillance	Daily review during 1 mo
Hayashino et al,^39^ 2012	Japan	2009	Several	836 (69.8)	Any career level	<39 (22.9)	7.9	Longitudinal	WHO-5 (<13)	Baseline	Self-reported	1 y after baseline
Kalmbach et al,^40^ 2017	United States	2012	Several	1215 (58.0)	Training physicians	Mean (SD): 27.5 (2.7)	48.9	Longitudinal	PHQ-9 (≥10)	Third and sixth mo of internship^b^	Self-reported	Third and sixth mo of internship
Kang et al,^41^ 2013	South Korea	2010	Several	86 (58.5)	Training physicians	<31 (77.9)	25.6	Cross-sectional	PRIME-MD-2 (yes to either item)	Single measure	Self-reported	Single measure
de Oliveira et al,^16^ 2013	United States	2011	Anesthesiology	1345 (54.4)^c^	Training physicians	<31 (53.8)	43.0	Cross-sectional	HANDS (≥9)	Single measure	Self-reported	Single measure
Sen et al,^14^ 2010	United States	2009	Several	740 (58.2)	Training physicians	<31 (85.7)	54.5	Longitudinal	PHQ-9 (≥10)	Preinternship + third, sixth, ninth, and twelfth mo of internship	Self-reported	Third, sixth, ninth, and twelfth mo of internship
Sen et al,^15^ 2013	United States	2007	Several	2323 (58.0)	Training physicians	Mean (SD): 27.5 (3.0)	50.9	Longitudinal	PHQ-9 (≥10)	Preinternship + third, sixth, ninth, and twelfth mo of internship	Self-reported	Third, sixth, ninth, and twelfth mo of internship
Shanafelt et al,^42^ 2010	United States	2008	Surgical	7905 (32.0)	Any career level	Median (IQR): 51 (43-59)	13.3	Cross-sectional	PRIME-MD-2 (yes to either item)	Single measure	Self-reported	Single measure
Tawfik et al,^43^ 2018	United States	2014	Several	6586 (19.2)	Any career level	Median (IQR): 56 (45-63)	32.9	Cross-sectional	PRIME-MD-2 (yes to either item)	Single measure	Self-reported	Single measure
West et al,^13^ 2009	United States	2003	Internal medicine	380 (88.4)	Training physicians	<31 (63.2)	37.9	Longitudinal	PRIME-MD-2 (yes to either item)	Every 6 mo from residency onset to completion	Self-reported	Every 3 mo from residency onset to completion
West et al,^44^ 2006^d^	United States	2003	Internal medicine	184 (84.0)^d^	Training physicians	<31 (70.1)	35.9	Longitudinal	PRIME-MD-2 (yes to either item)	Every 6 mo from residency onset to completion	Self-reported	Every 3 mo from residency onset to completion

Abbreviations: HANDS, Harvard National Depression Screening Day Scale; IQR, interquartile range; PHQ-9, 9-item Patient Health Questionnaire; PRIME-MD-2, 2-item Primary Care Evaluation of Mental Disorders; WHO-5, 5-item World Health Organization Well-being Index.

^a^ Number of participants included in the active surveillance of medical errors.

^b^ A baseline assessment of depressive symptoms was performed to exclude physicians with a positive screening for depression before internship onset.

^c^ Responses to the question, “I make mistakes with negative consequences to patients.”

^d^ Included only in the meta-analysis of medical errors associated with depressive symptoms. A more recent publication with a more comprehensive population (West et al^13^) reported on depressive symptoms associated with medical errors.

All but 1 study^12^ (9%) used self-report measures of medical errors. Eight studies (73%) inquired about medical errors in the past 3 months,^13,14,15,40,41,42,43,44^ 2 (18%) inquired about medical errors in the past year,^16,39^ and 1 (9%) actively surveyed medical errors in a 1-month interval.^12^ Assessment measures and definitions of medical errors adopted by individual studies are available in eTable 2 in the Supplement. Although most studies inquired about major or harmful medical errors,^13,14,15,16,39,40,42,43,44^ 1 study (9%) inquired whether physicians were concerned about errors of any type,^41^ and 1 study (9%) trained a team of nurses and physicians to collect daily reports of all medication errors occurring on wards and to actively review all medical records and medication orders using structured data forms.^12^ When evaluated by the established quality assessment criteria, 6 studies (55%) were considered as methodologically strong on the basis of design^12,13,14,15,39,40,44^; 8 (73%), on the basis of sample size^13,14,15,16,39,40,42,43^; 5 (46%), on the basis of ascertainment of depressive symptoms measure^12,14,15,16,40^; 8 (73%), on the basis of representativeness of the sample^12,14,15,16,39,40,42,43^; and all, on the basis of descriptive characteristics of participants.^12,13,14,15,16,39,40,41,42,43,44^ Detailed quality indicators for each study are available in eTable 3 in the Supplement.

Of the 11 included studies, 1 (9%) was used only in the meta-analysis of medical errors associated with subsequent depressive symptoms.^44^ The reason for excluding this study from the other analyses is that a more recent article reported data on depressive symptoms associated with subsequent medical errors in a more comprehensive sample of physicians.^13^ Because the more recent study did not report data on medical errors associated with subsequent depressive symptoms, the previous study was included in this directionality meta-analysis and excluded from all other analyses to avoid overlapping data. The approach recommended by Zhang and Yu^27^ was used for computing RR estimates in 2 studies that reported associations of depressive symptoms and medical errors in the format of an odds ratio.^13,44^

### Associations Between Depressive Symptoms and Medical Errors

**Figure 2.**
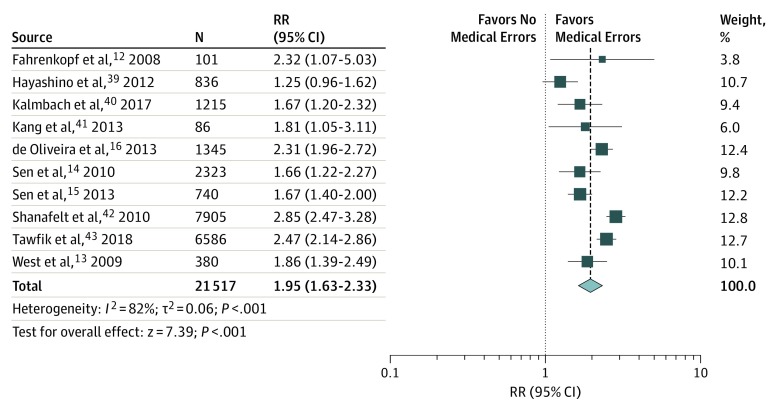
Meta-analysis of the Association Between Physician Depressive Symptoms and Medical Errors The size of squares is proportional to the weight of each study. Horizontal lines indicate the 95% CI of each study; diamond, the pooled estimate with 95% CI; N, the number of participants at baseline; and RR, relative risk.

Meta-analytic pooling of the associations between depressive symptoms and medical errors yielded a summary RR of 1.95 (95% CI, 1.63-2.33), with high heterogeneity across the studies (χ^2^ = 49.91; *P* < .001; *I*^2^ = 82%; τ^2^ = 0.06) (Figure 2). The sensitivity analysis, in which the meta-analysis was serially repeated after exclusion of each study, demonstrated that no individual study had an implication for the overall RR estimate of more than 0.12 points (these estimates varied from 1.85 [95% CI, 1.56-2.19] to 2.07 [95% CI, 1.77-2.43]) (eFigure 1 in the Supplement).

### Direction of the Associations 

**Figure 3.**
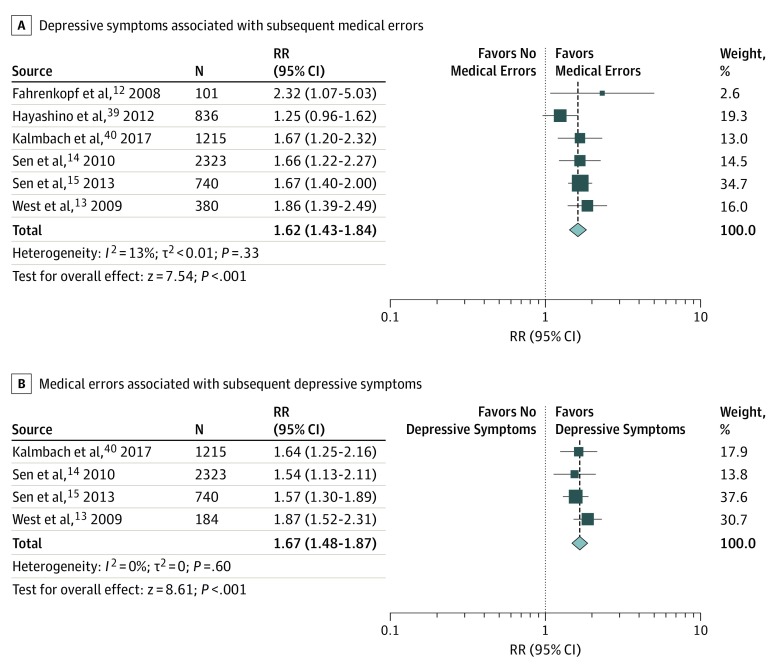
Meta-analyses of Long-term Studies of the Association Between Physician Depressive Symptoms and Medical Errors The size of squares is proportional to the weight of each study. Horizontal lines indicate the 95% CI of relative risk (RR) estimate in each study; diamonds, the pooled estimate with 95% CI; and N, the number of participants at baseline.

All of the 7 longitudinal studies included in the present review investigated the association of physician depressive symptoms in the next 1,^12^ 3,^13,14,15,40,44^ or 12 months.^39^ One study^44^ was removed from the first directionality analysis because a later publication, which included a more comprehensive sample, also reported on data regarding depressive symptoms associated with subsequent medical errors.^13^ Meta-analytic pooling of physician depression associated with medical errors resulted in a pooled RR of 1.62 (95% CI, 1.43-1.84), with low heterogeneity across studies (χ^2^ = 5.77; *P* = .33; *I*^2^ = 13%; τ^2^ < 0.01) (Figure 3).

Similarly, 4 of the 7 longitudinal studies provided data on medical errors associated with depressive symptoms in the next 3 months.^14,15,40,44^ Meta-analytic pooling of these 4 studies (involving 4462 physicians) resulted in a summary RR of 1.67 (95% CI, 1.48-1.87), with low heterogeneity across studies (χ^2^ = 1.85; *P* = .60; *I*^2^ = 0%; τ^2^ = 0), suggesting that the association between physician depression and medical errors is bidirectional (Figure 3).

### Associations Stratified by Study-Level Characteristics

To identify potential sources of heterogeneity, we performed subgroup meta-analysis of studies stratified by different study-level characteristics when at least 2 studies were available in each comparator subgroup. Studies with exclusively surgical specialties yielded a summary RR estimate that was significantly higher than the summary RR estimate in studies that also included nonsurgical specialties (2.59 [95% CI, 2.10-3.16] vs 1.79 [95% CI, 1.46-3.16]). Furthermore, US studies yielded higher estimates of the association between depression and medical errors compared with non-US studies (2.10 [95% CI, 1.77-2.46] vs 1.39 [95% CI, 1.00-1.93]). Summary RR estimates for studies assessing depressive symptoms through the HANDS or the PRIME-MD-2 were significantly higher compared with the ones identified through the PHQ-9 (HANDS: 2.32 [95% CI, 1.97-2.72]; PRIME-MD-2: 2.39 [95% CI, 1.97-2.86]; PHQ-9: 1.67 [95% CI, 1.45-1.92]) (eFigure 2 in the Supplement). No statistically significant differences in RR estimates were found between subgroups of studies stratified by physician career level or studies inquiring physicians about medical errors in the past 3 or 12 months.

A single study assessed depressive symptoms associated with medication errors actively surveyed in the next month.^12^ The sensitivity analysis that excluded this study did not show a significant reduction in heterogeneity statistics (from 1.95; 95% CI, 1.63-2.33; χ^2^ = 49.91; *P* < .001; *I*^2^ = 82%; τ^2^ = 0.06 to 1.94; 95% CI, 1.61-2.33; χ^2^ = 49.88; *P* < .001; *I*^2^ = 84%; τ^2^ = 0.06). In contrast, the sensitivity analysis that excluded the only study^39^ that used the WHO-5 to assess physician depressive symptoms resulted in a reduction in all heterogeneity statistics (from 1.95; 95% CI, 1.63-2.33; χ^2^ = 49.91; *P* < .001; *I*^2^ = 82%; τ^2^ = 0.06 to 2.07; 95% CI, 1.77-2.43; χ^2^ = 31.91; *P* < .001; *I*^2^ = 75%; τ^2^ = 0.04) (eFigure 1 in the Supplement). Metaregression results revealed that RR estimates did not significantly vary with baseline survey year (estimate = 0.01; 95% CI, –0.05 to 0.07; QM [statistic for the test of moderators] = 0.14; *P* = .71) or percentage of female physicians (estimate = –0.06; 95% CI, –1.13 to 1.00; QM = 0.01; *P* = .91) (eFigure 3 in the Supplement).

When evaluated by the quality assessment indicators, longitudinal studies yielded summary RR estimates that were significantly lower compared with those from the cross-sectional sectional studies (1.62; 95% CI, 1.43-1.84; χ^2^ = 5.77; *P* = .33; *I*^2^ = 13%; τ^2^ < 0.01 vs 2.51; 95% CI, 2.20-2.83; χ^2^ = 5.44; *P* = .14; *I*^2^ = 45%; τ^2^ < 0.01). No statistically significant differences in RR estimates were found between subgroups of studies stratified by sample size, ascertainment of the depression measure, representativeness of the sample, or descriptive characteristics of the participants (eFigure 4 in the Supplement).

### Assessment of Publication Bias

A funnel plot of studies that reported on physician depressive symptoms associated with medical errors is presented in eFigure 5 in the Supplement). The Egger test indicated the absence of significant publication bias (intercept = –2.79; *P* = .12).

## Discussion

This systematic review and meta-analysis of 11 studies involving 21 517 physicians demonstrated an association between physician depressive symptoms and an increased risk for perceived medical errors (RR, 1.95; 95% CI, 1.63-2.33). We also found that the magnitude of the associations of physician depressive symptoms and perceived medical errors were relatively consistent across studies that assessed training and practicing physicians, providing additional evidence that physician depression has implications for the quality of care delivered by physicians at different career stages.

Subgroup meta-analysis of studies stratified by different study-level characteristics identified study design, specialty type, geographic region, and depressive symptoms measure as possible sources of heterogeneity in this meta-analysis. The 6 longitudinal studies that assessed physician depressive symptoms associated with subsequent medical errors yielded a significantly lower summary RR estimate compared with the 4 cross-sectional studies included in this meta-analysis (1.62 [95% CI, 1.43-1.84] vs 2.51 [95% CI, 2.20-2.83]), but a significant increased risk for medical errors among physicians with depressive symptoms was identified in both study designs.

Similarly, although the summary RR estimates for studies that included nonsurgical specialties, that were from non-US countries, and that used the PHQ-9 as a measure of depressive symptoms were significantly lower than the summary RR estimates identified for their reference subgroups, the estimates were still statistically significant for all analyzed subgroups. These results support the main finding that depressive symptoms are associated with an increased risk for medical errors among physicians.

In line with these results, sensitivity analysis demonstrated that no individual study was associated with the overall RR estimate by more than 0.12 points (overall RR estimates in sensitivity analysis varied from 1.85 [95% CI, 1.56-2.19] to 2.07 [95% CI, 1.77-2.43]). The study that accounted for the largest variation in the magnitude of RR estimates (from 1.95 [95% CI, 1.63-2.33] to 2.07 [95% CI, 1.77-2.43]) used the WHO-5 for the ascertainment of depression in Japanese physicians.^39^ The WHO-5 was originally designed as a measure of subjective well-being and has been validated as a depression screening instrument.^45^ Studies conducted in primary care settings have suggested that the WHO-5′s broad statements tend to favor sensitivity at the cost of specificity when screening for depression in the general population,^46,47,48,49^ which might have been a source of heterogeneity in the present study.

A previous meta-analysis has associated physician burnout and emotional distress with patient safety outcomes.^50^ The present meta-analysis advances the findings of this past work in different ways. First, the issue of quantifying heterogeneous constructs of emotional distress in the same meta-analysis was overcome by focusing on depressive symptoms, which have well-established clinical criteria and methods of assessment.^20,51^ Similarly, by working with RR instead of odds ratio estimates, we were able to more accurately estimate the magnitude of the association between depressive symptoms and perceived medical errors.^52,53^ Furthermore, the analysis of 7 longitudinal studies^12,13,14,15,39,40,44^ allowed us to demonstrate that physician depressive symptoms are associated with future medical errors (RR, 1.62; 95% CI, 1.43-1.84; n = 5595 physicians from 6 studies^12,13,14,15,39,40^) and that medical errors are associated with future depressive symptoms in physicians (RR, 1.67; 95% CI, 1.48-1.87; n = 4462 physicians from 4 studies^14,15,40,44^). Taken together, these data suggest that the association between physician depression and medical errors is bidirectional. To our knowledge, this study is the first to systematically review the direction of the associations between physician depressive symptoms and medical errors.

Studies have recommended the addition of physician well-being to the Triple Aim of enhancing the patient experience of care, improving the health of populations, and reducing the per capita cost of health care.^54,55,56,57^ Results of the present study endorse the Quadruple Aim movement by demonstrating not only that medical errors are associated with physician health but also that physician depressive symptoms are associated with subsequent errors. Given that few physicians with depression seek treatment^58,59^ and that recent evidence has pointed to the lack of organizational interventions aimed at reducing physician depressive symptoms,^25^ our findings underscore the need for institutional policies to remove barriers to the delivery of evidence-based treatment to physicians with depression. Investments in patient safety have been associated with significant reductions in health care costs,^60^ and the bidirectional associations between physician depressive symptoms and perceived medical errors verified by this meta-analysis suggest that physician well-being is critical to patient safety. Further studies are needed to explore these associations. Such research should investigate whether systematic interventions for reducing depressive symptoms could be factors in decreased medical errors.

### Limitations

This systematic review and meta-analysis has some limitations. First, 10 of 11 studies included relied on self-report measures of medical errors.^13,14,15,16,39,40,41,42,43,44^ Although substantial differences in RR estimates and heterogeneity statistics were not identified by sensitivity analysis that removed the only study that assessed medical errors through active surveillance,^12^ the small sample size of the referred study limited its weight in the overall meta-analysis. Furthermore, although self-reported errors have been found to be highly correlated with recorded events,^61^ the self-report nature of the included studies may have introduced bias to the present results. For instance, physicians with depression may be more likely to perceive medical errors, which may drive the association between depressive symptoms and medical errors. However, the secondary meta-analyses of longitudinal studies that assessed depressive symptoms associated with subsequent medical errors and medical errors associated with future depressive symptoms demonstrated significantly increased risk estimates, which suggests the existence of bidirectional temporal associations between physician depressive symptoms and perceived medical errors. Similarly, all included studies examined and ascertained depressive symptoms from self-report inventories that varied in sensitivity and specificity. Therefore, the results demonstrated the presence of associations between depressive symptoms and perceived medical errors rather than the association between a clinical diagnosis of depression and medical errors.

Second, the 10 studies that evaluated self-reported medical errors included general questions about either major,^13,14,15,39,40,42,43,44^ harmful,^16^ or any^41^ medical errors. By doing so, these studies might have underestimated particular acts and omissions with potential to harm that physicians might not have considered to be a major, harmful, or any medical error. In the only study that assessed errors through active surveillance, more than 60% of the observed medical errors were considered to be potentially harmful,^12^ which suggests that a large portion of medical errors committed by physicians could have negative consequences for patients.

Third, the small number of studies included in some of the subgroups may have biased some of the subgroup analysis results.^62^ Fourth, despite the significant overall effect of the meta-analytic model of medical errors associated with subsequent depressive symptoms, few studies (4 studies with 4462 physicians)^14,15,40,44^ were included in this directional analysis, which might also have introduced bias to the results. Fifth, most studies (9 of 11) assessed US physicians.^12,13,14,15,16,40,42,43,44^ Therefore, the results may not be generalizable to physicians in other countries.

Sixth, although the 3 studies that evaluated both practicing and training physicians included the largest number of physicians in this meta-analysis (15 327 of 21 517),^39,42,43^ most of the included studies (8 of 11) exclusively assessed populations of training physicians.^12,13,14,15,16,40,41,44^ Although the subgroup meta-analysis that stratified studies by physician career level did not identify significant differences between the 2 subgroups, generalizations of the present study results to populations of practicing physicians should be done with caution. Seventh, all references included were from full-text articles published in peer-reviewed journals. Although no evidence of publication bias was verified by Egger test, the exclusion of unpublished data and gray literature might have introduced selection bias to this analysis.

## Conclusions

By combining data from multiple studies, this systematic review and meta-analysis found that physician depressive symptoms were associated with increased risk for perceived medical errors and that the association between depressive symptoms and perceived errors was bidirectional. Future research is needed to evaluate the associations of physician depressive symptoms with objective measures of medical errors, such as active surveillance. Studies that include physicians from different countries could answer whether cultural and socioeconomic aspects play a role in the associations between depressive symptoms and errors. Future research is also needed into the degree to which interventions for reducing physician depressive symptoms could mitigate medical errors and improve physician well-being and patient care.

## Notes

Supplement. **eMethods.** Syntaxes Used in Database Searches **eTable 1.** Sensitivities and Specificities of Commonly Used Instruments With Cutoff Scores Adopted by Individual Studies Included in This Meta-analysis **eTable 2.** Medical Errors Measurements Adopted by Individual Studies **eTable 3.** Detailed Quality Assessment **eFigure 1.** Sensitivity Analysis **eFigure 2.** Subgroup Meta-analyses Stratified by Study-Level Characteristics **eFigure 3.** Bubble Plots Displaying Meta-Regression Results **eFigure 4.** Meta-analyses of the Associations Between Physician Depressive Symptoms and Medical Errors Stratified by Study Quality Indicators **eFigure 5.** Funnel Plot **eReferences.**
