# Supplementary material for: Association Between Physician Depressive Symptoms and Medical Errors: A Systematic Review and Meta-analysis
Source: JAMA Netw Open. 2019 Nov 27;2(11):e1916097. doi: 10.1001/jamanetworkopen.2019.16097 (PMC6902829; doi:10.1001/jamanetworkopen.2019.16097)
Supplement: Supplement. — eMethods. Syntaxes Used in Database Searches eTable 1. Sensitivities and Specificities of Commonly Used Instruments With Cutoff Scores Adopted by Individual Studies Included in This Meta-analysis eTable 2. Medical Errors Measurements Adopted by Individual Studies eTable 3. Detailed Quality Assessment eFigure 1. Sensitivity Analysis eFigure 2. Subgroup Meta-analyses Stratified by Study-Level Characteristics eFigure 3. Bubble Plots Displaying Meta-Regression Results eFigure 4. Meta-analyses of the Associations Between Physician Depressive Symptoms and Medical Errors Stratified by Study Quality Indicators eFigure 5. Funnel Plot eReferences. [file jamanetwopen-2-e1916097-s001.pdf]

Supplementary Online Content

Pereira-Lima K, Mata DA, Loureiro SR, Crippa JA, Bolsoni LM, Sen S. Association between physician depressive symptoms and medical errors: a systematic review and meta-analysis. *JAMA Netw Open.* 2019;2(11):e1916097. doi:10.1001/jamanetworkopen.2019.16097

eMethods. Syntaxes Used in Database Searches

eTable 1. Sensitivities and Specificities of Commonly Used Instruments With Cutoff Scores Adopted by Individual Studies Included in This Meta-analysis

eTable 2. Medical Errors Measurements Adopted by Individual Studies

eTable 3. Detailed Quality Assessment

eFigure 1. Sensitivity Analysis

eFigure 2. Subgroup Meta-analyses Stratified by Study-Level Characteristics

eFigure 3. Bubble Plots Displaying Meta-Regression Results

eFigure 4. Meta-analyses of the Associations Between Physician Depressive

Symptoms and Medical Errors Stratified by Study Quality Indicators

eFigure 5. Funnel Plot

eReferences.

This supplementary material has been provided by the authors to give readers additional information about their work.

eMethods. Syntaxes Used in Database Searches

Database	Search strategy	Results
Embase	(depressed OR depression OR 'major depression' OR 'major depressive disorder' OR mdd OR sadness OR suicid* OR suicide) AND (accident OR 'diagnostic error' OR error* OR 'inappropriate prescribing' OR malpractice OR 'medical error' OR mistake* OR mismanagement OR 'medication error' OR 'patient harm') AND (doctor OR doctors OR fellow* OR 'house staff' OR intern OR interns OR internship OR 'medical education' OR 'medical residency' OR 'medical resident*' OR 'medical trainee*' OR physician* OR 'practicing physician*' OR 'residency training' OR 'resident physician*') AND [0000-2018]/py	3,372 references
ERIC	[illegible] (suicid*) OR (suicide)) AND ((accident) OR ('diagnostic error') OR (error*) OR ('inappropriate prescribing') OR (malpractice) OR ('medical error') OR [illegible] (mismanagement) OR ('medication error') OR ('patient harm')) AND ((doctor) OR (doctors) OR (fellow*) OR ('house staff') OR (intern) OR (interns) [illegible]p) OR ('medical education') OR ('medical residency') OR ('medical resident*') OR ('medical trainee*') OR (physician*) OR ('practicing physician*') OR [illegible]ining') OR ('resident physician*')) Additional filters: before date 1/1/2019	18 references
PsycINFO	"((Depressed) OR (depression) OR ('major depression') OR ('major depressive disorder') OR (MDD) OR (sadness) OR (suicid*) OR (suicide)) AND ((accident) OR ('diagnostic error') OR (error*) OR ('inappropriate prescribing') OR (malpractice) OR ('medical error') OR (mistake*) OR (mismanagement) OR ('medication error') OR ('patient harm')) AND ((doctor) OR (doctors) OR (fellow*) OR ('house staff') OR (intern) OR (interns) OR (internship) OR ('medical education') OR ('medical residency') OR ('medical resident*') OR ('medical trainee*') OR (physician*) OR ('practicing physician*') OR ('residency training') OR ('resident physician*')) Limits: published date: -2018/12/31	641 references
Pubmed	((depressed) OR (depression) OR (Depression[MeSH]) OR (Depressive disorder[MeSH]) OR (Depressive disorder, major[MeSH]) OR (Major depression) OR (Major depressive disorder) OR (MDD) OR (sadness) OR (suicid*) OR (Suicide[MeSH])) AND ((accident) OR (Diagnostic Errors[MeSH]) OR (error*) OR (inappropriate prescribing) OR (Malpractice[MeSH]) OR (Medical Errors[MeSH]) OR (mistake*) OR (mismanagement) OR (Medication Errors[MeSH]) OR (Patient Harm[MeSH])) AND ((doctor) OR (doctors) OR (Education, medical[MeSH]) OR (fellow*) OR ("house staff") OR (intern) OR (interns) OR (internship) OR (medical residency) OR (medical resident*) OR (medical trainee*) OR (Physician*) OR (Physicians[MeSH]) OR (practicing physician*) OR (residency training) OR (resident physician*)) AND ("0000/01/01"[Date - Publication] : "2018/12/31"[Date - Publication])	1,598 references
Scopus	(TITLE-ABS-KEY ((depressed) OR (depression) OR ("major depression") OR ("major depressive disorder") OR (mdd) OR (sadness) OR (suicid*) OR (suicide)) AND TITLE-ABS-KEY ((accident) OR ("diagnostic error") OR (error*) OR ("inappropriate prescribing") OR (malpractice) OR ("medical error") OR (mistake*) OR (mismanagement) OR ("medication error") OR ("patient harm")) AND TITLE-ABS-KEY (doctor OR doctors OR fellow* OR "house staff" OR intern OR interns OR internship OR "medical education" OR "medical residency" OR "medical resident*" OR "medical trainee*" OR "physician*" OR "practicing physician*" OR "residency training*" OR "resident physician*")) AND PUBYEAR < 2019	2,368 references
Web of Science	TOPIC: ((((Depressed) OR (depression) OR ('major depression') OR ('major depressive disorder') OR (MDD) OR (sadness) OR (suicid*) OR (suicide)) AND ((accident) OR ('diagnostic error') OR (error*) OR ('inappropriate prescribing') OR (malpractice) OR ('medical error') OR (mistake*) OR (mismanagement) OR ('medication error') OR ('patient harm')) AND ((doctor) OR (doctors) OR (fellow*) OR ('house staff') OR (intern) OR (interns) OR (internship) OR ('medical education') OR ('medical residency') OR ('medical resident*') OR ('medical trainee*') OR (physician*) OR ('practicing physician*') OR ('residency training') OR ('resident physician*')))) *Indexes=SCI-EXPANDED, SSCI, A&HCI, CPCI-S, CPCI-SSH, BKCI-S, BKCI-SSH, ESCI, CCR-EXPANDED Timespan=1900-2018*	1,001 references

©2019 Pereira-Lima K et al. *JAMA Network Open.*

eTable 1. Sensitivities and Specificities of Commonly Used Instruments With Cutoff Scores Adopted by Individual Studies Included in This Meta-analysis

First author, year	Cutoff adopted by individual studies	Sensitivity	Specificity
Harvard national depression screening day scale (HANDS)[1]			
Fahrenkopf, 2008[2]	≥ 9	95%	94%
Oliveira, 2013[3]			
Patient Health Questionnaire-9 (PHQ-9)[4]			
Kalmbach, 2017[5]	≥ 10	88%	88%
Sen, 2010[6]			
Sen, 2013[7]			
Primary Care Evaluation of Mental Disorders (PRIME-MD)[8]			
Kang, 2013[9]	≥ 1	91%	66%
Shanafelt, 2010[10]			
Tawfik, 2018[11]			
West, 2009[12]			
West, 2006[13]			
World Health Organization-Five Well-Being Index (WHO-5)[14]			
Hayashino, 2012[15]	≥ 13	90%	63%

eTable 2. Medical Errors Measurements Adopted by Individual Studies

First author, year	Method of assessment	Survey question or surveillance methodology	Error definition
Fahrenkopf, 2008[2]	Active surveillance	A team of trained nurses and physicians collected daily reports of all medication errors that occurred on studied wards from clinical staff and reviewed all charts and medication orders using structured data forms (one-month period)	"Any error in the ordering, transcription, or administration of a medication, whether harmful or trivial"
Hayashino, 2012[15]	Self-report (Survey)	"Are you concerned that you have made any major medical mistakes in the last year?" (Response options: "yes", "no")	"Yes" to the medical error question
Kalmbach, 2017[5]	Self-report (Survey)	"Are you concerned you have made any major medical errors in the last 3 months?" (Response options: "yes", "no")	"Yes" to the medical error question
Kang, 2013[9]	Self-report (Survey)	"Have you committed a medical error in the last 3 months?" (Response options: "yes", "no")	"Yes" to the medical error question
Oliveira, 2013[3]	Self-report (Survey)	"I make mistakes with negative consequences to my patients" (Response options: "never", "rarely", "sometimes", "often", "always")	"Sometimes", "Often", or "Always" to the medical error question
Sen, 2010[6]	Self-report (Survey)	"Are you concerned you have made any major medical errors in the last 3 months?" (Response options: "yes", "no")	"Yes" to the medical error question
Sen, 2013[7]	Self-report (Survey)	"Are you concerned you have made any major medical errors in the last 3 months?" (Response options: "yes", "no")	"Yes" to the medical error question
Shanafelt, 2010[10]	Self-report (Survey)	"Are you concerned you have made any major medical error in the last 3 months?" (Response options: "yes", "no")	"Yes" to the medical error question
Tawfik, 2018[11]	Self-report (Survey)	"Are you concerned you have made any major medical error in the last 3 months?" (Response options: "yes", "no")	"Yes" to the medical error question
West, 2009[12]	Self-report (Survey)	"Are you concerned you have made any major medical error in the last 3 months?" (Response options: "yes", "no")	"Yes" to the medical error question
West, 2006[13]	Self-report (Survey)	"Are you concerned you have made any major medical error in the last 3 months?" (Response options: "yes", "no")	"Yes" to the medical error question

©2019 Pereira-Lima K et al. *JAMA Network Open.*

eTable 3. Detailed Quality Assessment

First author, year	Design	Sample Size	Ascertainment – depressive symptoms	Representativeness	Descriptive data
Fahrenkopf, 2008[2]	Longitudinal (Strong)	< 200 (Weak)	Sn & Sp ≥ 75% (Strong)	≥2 sites (Strong)	More descriptive (Strong)
Hayashino, 2012[15]	Longitudinal (Strong)	≥ 200 (Strong)	Sn or Sp < 75% (Weak)	≥2 sites (Strong)	More descriptive (Strong)
Kalmbach, 2017[5]	Longitudinal (Strong)	≥ 200 (Strong)	Sn & Sp ≥ 75% (Strong)	≥2 sites (Strong)	More descriptive (Strong)
Kang, 2013[9]	Cross-sectional (Weak)	< 200 (Weak)	Sn or SP < 75% (Weak)	< 2 sites (Weak)	More descriptive (Strong)
Oliveira, 2013[3]	Cross-sectional (Weak)	≥ 200 (Strong)	Sn & Sp ≥ 75% (Strong)	≥2 sites (Strong)	More descriptive (Strong)
Sen, 2010[6]	Longitudinal (Strong)	≥ 200 (Strong)	Sn & Sp ≥ 75% (Strong)	≥2 sites (Strong)	More descriptive (Strong)
Sen, 2013[7]	Longitudinal (Strong)	≥ 200 (Strong)	Sn & Sp ≥ 75% (Strong)	≥2 sites (Strong)	More descriptive (Strong)
Shanafelt, 2010[10]	Cross-sectional (Weak)	≥ 200 (Strong)	Sn or Sp < 75% (Weak)	≥2 sites (Strong)	More descriptive (Strong)
Tawfik, 2018[11]	Cross-sectional (Weak)	≥ 200 (Strong)	Sn or Sp < 75% (Weak)	≥2 sites (Strong)	More descriptive (Strong)
West, 2009[12]	Longitudinal (Strong)	≥ 200 (Strong)	Sn or Sp < 75% (Weak)	< 2 sites (Weak)	More descriptive (Strong)
West, 2006[13]	Longitudinal (Strong)	< 200 (Weak)	Sn or Sp < 75% (Weak)	< 2 sites (Weak)	More descriptive (Strong)

Abbreviations: Sn = Sensitivity; Sp = Specificity.

©2019 Pereira-Lima K et al. *JAMA Network Open.*

eFigure 1. Sensitivity Analysis

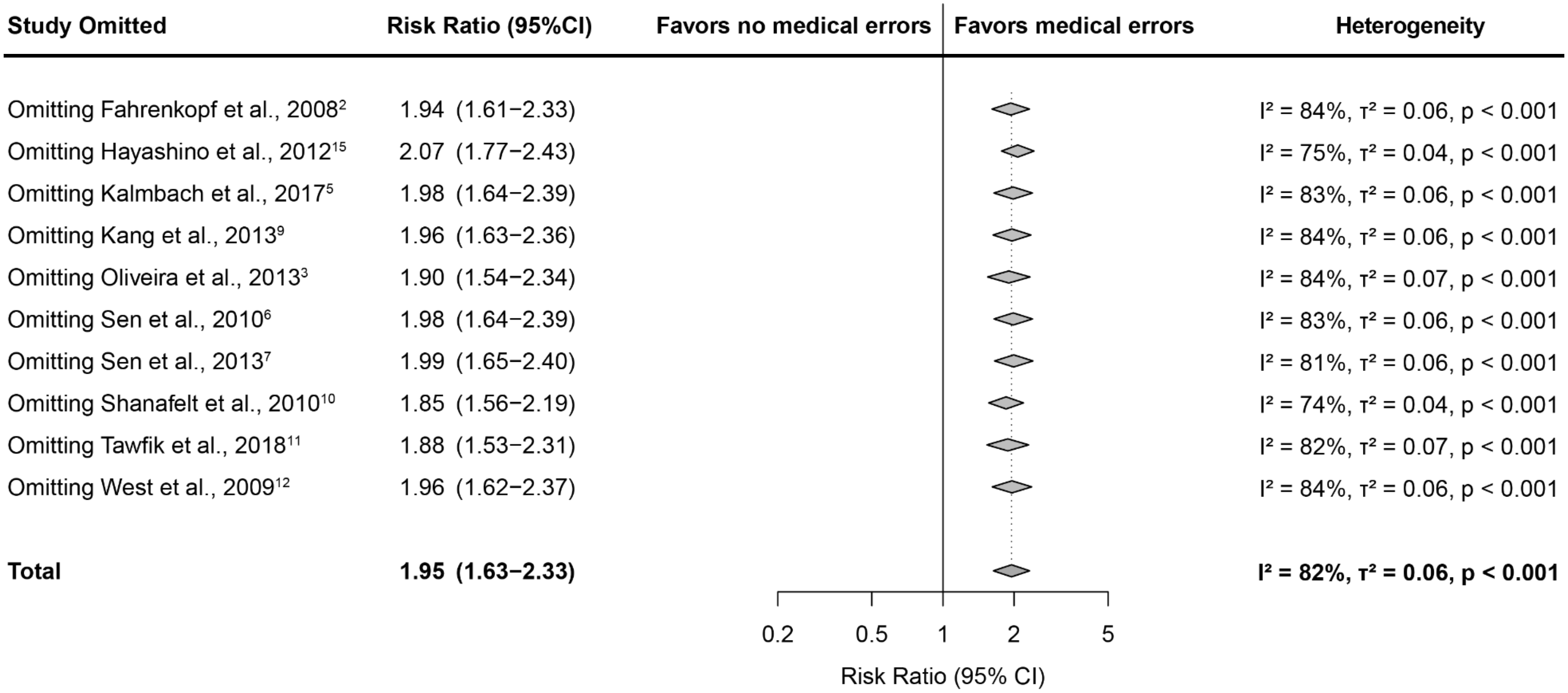

Legend: Studies are ordered alphabetically by first author. Summary estimates were calculated omitting one study at a time using a random effects model. N is the number of participants at baseline.

©2019 Pereira-Lima K et al. *JAMA Network Open.*

eFigure 2. Subgroup Meta-analyses Stratified by Study-Level Characteristics

Subgroup	Number of Studies	N	Interaction P–value	Risk Ratio (95% CI)	Heterogeneity
**Physician career level**					
Only training physicians	7	6,190	0.59	1.88 (1.65–2.14)	$I^2 = 33\%$, $\tau^2 < 0.01$, $p = 0.177$
Any professional level	3	15,327		2.10 (1.43–3.10)	$I^2 = 93\%$, $\tau^2 = 0.11$, $p < 0.001$
**Specialties**					
Nonsurgical and surgical	8	12,267	0.01	1.79 (1.46–2.16)	$I^2 = 73\%$, $\tau^2 = 0.05$, $p < 0.001$
Surgical only	2	9,250		2.59 (2.10–3.16)	$I^2 = 72\%$, $\tau^2 = 0.02$, $p = 0.058$
**Medical errors question**					
12 months	2	2,181	0.62	1.72 (0.93–3.13)	$I^2 = 93\%$, $\tau^2 = 0.18$, $p < 0.001$
3 months	7	19,235		2.01 (1.65–2.46)	$I^2 = 81\%$, $\tau^2 = 0.05$, $p < 0.001$
**Geographic location**					
US	8	20,595	0.03	2.10 (1.77–2.46)	$I^2 = 78\%$, $\tau^2 = 0.04$, $p < 0.001$
Non–US	2	922		1.39 (1.00–1.93)	$I^2 = 31\%$, $\tau^2 = 0.02$, $p = 0.228$
**Depressive symptoms measure**					
HANDS	2	1,446	< 0.01	2.32 (1.97–2.72)	$I^2 = 00\%$, $\tau^2 = 0.00$, $p = 0.986$
PHQ–9	3	4,278		1.67 (1.45–1.92)	$I^2 = 00\%$, $\tau^2 = 0.00$, $p = 0.998$
PRIME–MD	4	14,957		2.39 (1.97–2.86)	$I^2 = 65\%$, $\tau^2 = 0.02$, $p = 0.038$

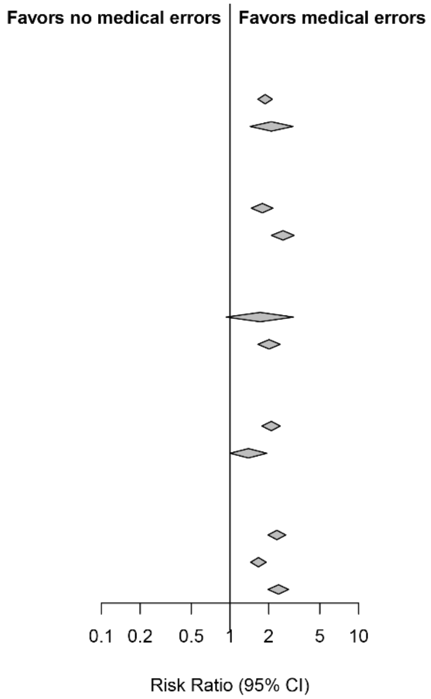

Diamonds indicate pooled estimates with 95% confidence intervals. N is the number of participants at baseline.

©2019 Pereira-Lima K et al. *JAMA Network Open.*

eFigure 3. Bubble Plots Displaying Meta-Regression Results

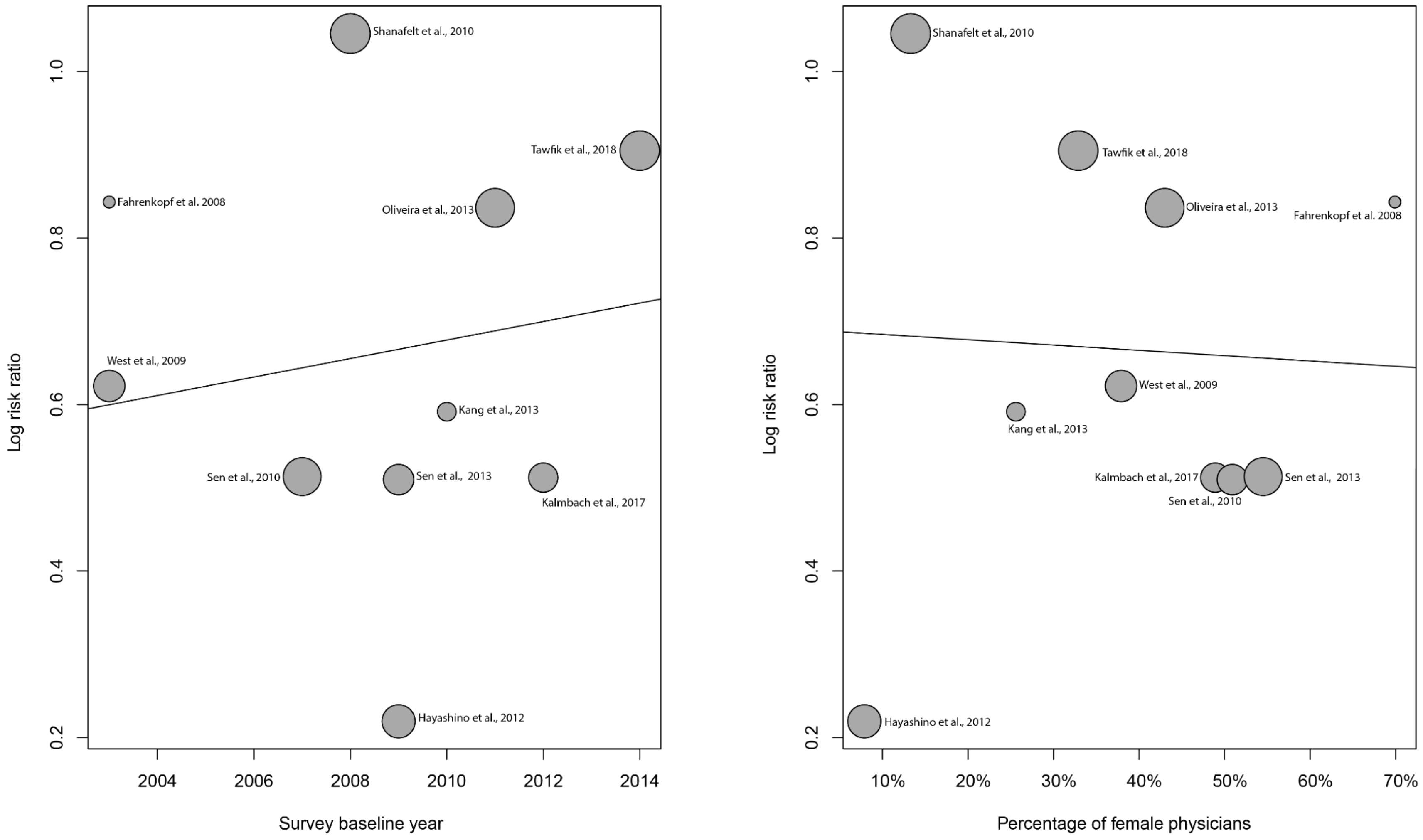

Meta-regression results for baseline year (*P*, .71) and percentage of female physicians (*P*, .91)

©2019 Pereira-Lima K et al. *JAMA Network Open.*

eFigure 4. Meta-analyses of the Associations Between Physician Depressive Symptoms and Medical Errors Stratified by Study Quality Indicators

Subgroup	Number of Studies	N	Interaction P−value	Risk Ratio (95% CI)	Heterogeneity
Design					
Longitudinal	6	5,595	< 0.01	1.62 (1.43−1.84)	$I^2 = 13\%, \tau^2 < 0·01, p = 0·329$
Cross−sectional	4	15,922		2.51 (2.20−2.83)	$I^2 = 45\%, \tau^2 < 0·01, p = 0·143$
Sample Size					
< 200	2	187	0.97	1.97 (1.26−3.06)	$I^2 = 00\%, \tau^2 = 0·00, p = 0·601$
≥ 200	8	21,330		1.93 (1.60−2.36)	$I^2 = 86\%, \tau^2 = 0·06, p < 0·001$
Ascertainment					
More valid	5	5,724	0.67	1.88 (1.57−2.25)	$I^2 = 55\%, \tau^2 = 0·02, p = 0·064$
Less valid	5	15,793		2.01 (1.51−2.69)	$I^2 = 88\%, \tau^2 = 0·09, p < 0·001$
Representativeness					
More representative	8	21,051	0.71	1.97 (1.62−2.41)	$I^2 = 86\%, \tau^2 = 0·07, p < 0·001$
Less representative	2	466		1.86 (1.43−2.39)	$I^2 = 00\%, \tau^2 = 0·00, p = 0·922$

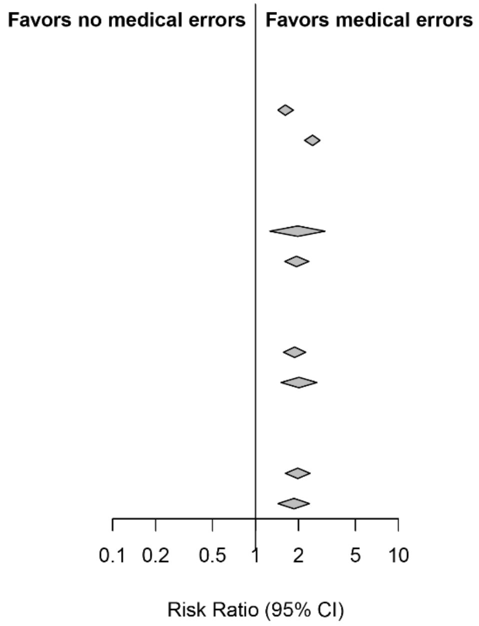

Diamonds indicate pooled estimates with 95% confidence intervals. N is the number of participants at baseline.

©2019 Pereira-Lima K et al. *JAMA Network Open.*

eFigure 5. Funnel Plot

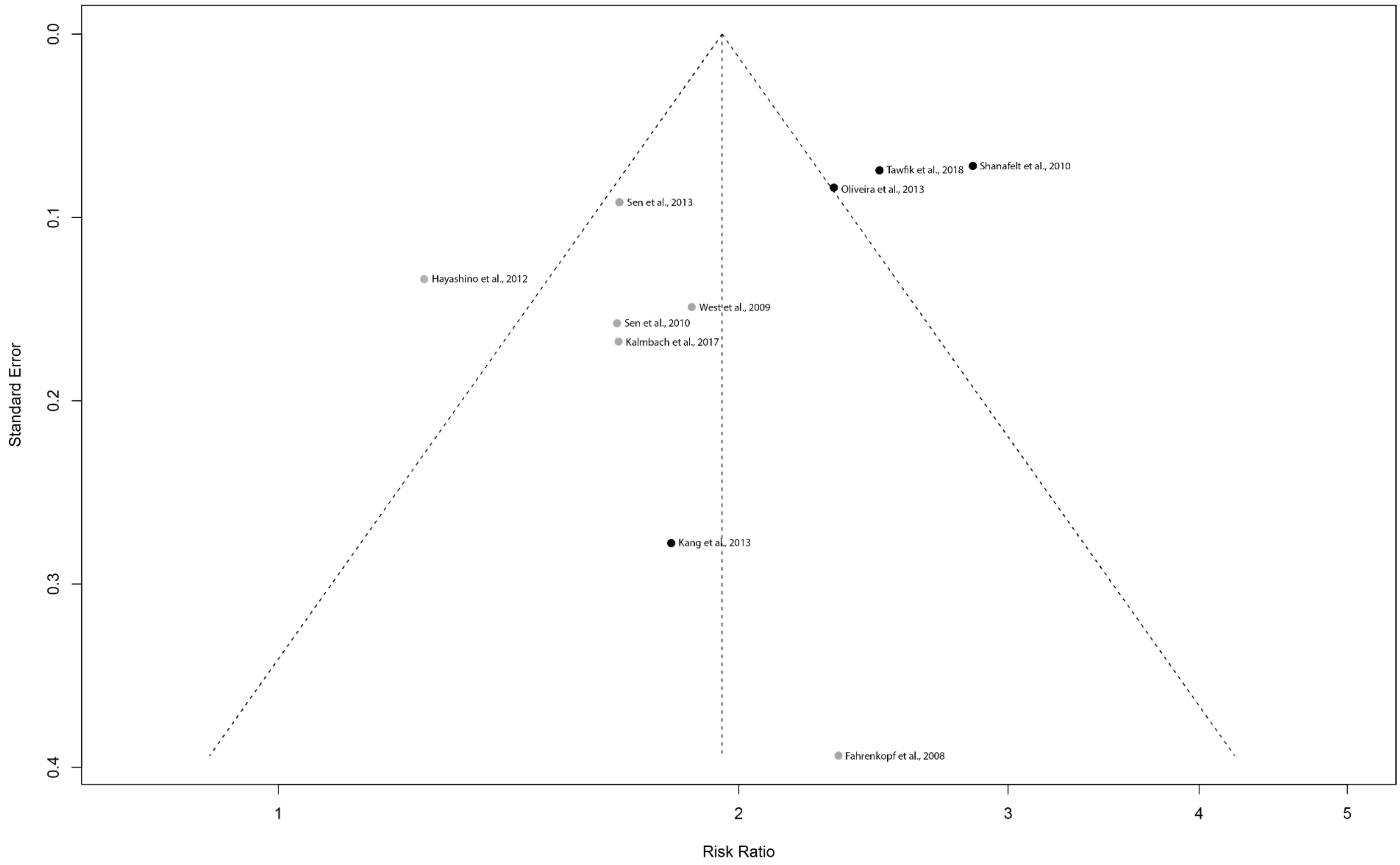

Funnel plot with pseudo 95% confidence intervals. Black dots represent cross-sectional studies. Gray dots represent longitudinal studies. Egger's test *P* = .12

©2019 Pereira-Lima K et al. *JAMA Network Open.*

eReferences

1. Baer L, Jacobs DG, Meszler-Reizes J, et al. Development of a brief screening instrument: the HANDS. *Psychother Psychosom.* 2000;69(1):35-41.
2. Fahrenkopf AM, Sectish TC, Barger LK, et al. Rates of medication errors among depressed and burnt out residents: prospective cohort study. *BMJ.* 2008;336(7642):488-491.
3. Oliveira GS, Chang R, Fitzgerald PC, et al. The prevalence of burnout and depression and their association with adherence to safety and practice standards: a survey of United States anesthesiology trainees. *Anesth Analg.* 2013;117(1):182-193.
4. Kroenke K, Spitzer RL, Williams JBW. The PHQ-9: validity of a brief depression severity measure. *J Gen Intern Med.* 2001;16(9):606-613.
5. Kalmbach DA, Arnedt JT, Song PX, Guille C, Sen S. Sleep disturbance and short sleep as risk factors for depression and perceived medical errors in first-year residents. *Sleep.* 2017;40(3):zsw073.
6. Sen S, Kranzler HR, Krystal JH, et al. A prospective cohort study investigating factors associated with depression during medical internship. *Arch Gen Psychiatry.* 2010;67(6):557-565.
7. Sen S, Kranzler HR, Didwania AK, et al. Effects of the 2011 duty hour reforms on interns and their patients: a prospective longitudinal cohort study. *JAMA Intern Med.* 2013;173(8):657-662.
8. Spitzer RL, Kroenke K, Linzer M, et al. Health-related quality of life in primary care patients with mental disorders: results from the PRIME-MD 1000 study. *Jama.* 1995;274(19):1511-1517.
9. Kang E-K, Lihm H-S, Kong E-H. Association of intern and resident burnout with self-reported medical errors. *Korean J Fam Med.* 2013;34(1):36-42.
10. Shanafelt TD, Balch CM, Bechamps G, et al. Burnout and medical errors among American surgeons. *Ann Surg.* 2010;251(6):995-1000.
11. Tawfik DS, Profit J, Morgenthaler TI, et al. Physician Burnout, Well-being, and Work Unit Safety Grades in Relationship to Reported Medical Errors. *Mayo Clin Proc.* 2018;93(11):1571-1580.
12. West CP, Tan AD, Habermann TM, Sloan JA, Shanafelt TD. Association of resident fatigue and distress with perceived medical errors. *JAMA.* 2009;302(12):1294-1300.
13. West CP, Huschka MM, Novotny PJ, et al. Association of perceived medical errors with resident distress and empathy: a prospective longitudinal study. *JAMA.* 2006;296(9):1071-1078.
14. Mergl R, Seidscheck I, Allgaier AK, Möller HJ, Hegerl U, Henkel V. Depressive, anxiety, and somatoform disorders in primary care: prevalence and recognition. *Depress Anxiety.* 2007;24(3):185-195.
15. Hayashino Y, Utsugi-Ozaki M, Feldman MD, Fukuhara S. Hope modified the association between distress and incidence of self-perceived medical errors among practicing physicians: prospective cohort study. *PLoS One.* 2012;7(4):e35585.

©2019 Pereira-Lima K et al. *JAMA Network Open.*
